# Multifunctional BiF_3_:Ln^3+^ (Ln = Ho, Er, Tm)/Yb^3+^ nanoparticles: an investigation on the emission color tuning, thermosensitivity, and bioimaging†

**DOI:** 10.1039/c9ra01018a

**Published:** 2019-04-08

**Authors:** Xinxin Yan, Tiesheng Li, Linna Guo, Honglei Li, Penglei Chen, Minghua Liu

**Affiliations:** College of Chemistry and Molecular Engineering, Zhengzhou University, The Key Lab of Chemical Biology and Organic Chemistry of Henan Province, The Key Lab of Nano-information Materials of Zhengzhou Zhengzhou 450001 P. R. China lts34@zzu.edu.cn; Beijing National Laboratory for Molecular Science, Institute of Chemistry, Chinese Academy of Sciences Beijing 100190 P. R. China

## Abstract

Pure cubic phase and uniform BiF_3_:Ln^3+^ (Ln = Ho, Er, Tm)/Yb^3+^ nanoparticles (NPs) were prepared by coprecipitation. The growth mechanism of BiF_3_:2%Er^3+^/20%Yb^3+^ NPs was proposed based on evolution analysis of the time-dependent morphology, in which BiF_3_:2%Er^3+^/20%Yb^3+^ was formed through the growth process of “nucleation to crystallization and Ostwald ripening”. The upconversion luminescence (UCL) properties and mechanism of BiF_3_:Ln^3+^ (Ln = Ho, Er, Tm)/Yb^3+^ under dual-wavelength excitation were also systematically investigated. The emission intensity of BiF_3_:2%Er^3+^/20%Yb^3+^ by dual-wavelength excitation (*λ* = 980 nm + 1550 nm) was 1.49 times more than that excited by 1550 nm or 980 nm individually. Furthermore, the properties of the bright white and multicolor UCL showed that yellow, purple, green, or pinkish light could be observed by controlling the doping concentration of Ln^3+^ (Ln = Yb, Er, Tm, and Ho), indicating that they had potential applications in backlight sources of color displays and security labeling. The temperature sensitivity of BiF_3_:2%Er^3+^/20%Yb^3+^ exhibited a downward tendency and its max value was about 0.0036 K^−1^ at 273 K. Cell toxicity tests showed that the UCNPs in phospholipid aqueous solution presented low cytotoxicity. Also, *in vivo* imaging and X-ray imaging revealed that the BiF_3_:2%Er^3+^/20%Yb^3+^ NPs had deep penetration and high contrast, which meant it could be used as a potential probe and contrast agent in *in vivo* optical bioimaging.

## Abstract 2

Multifunctional BiF_3_:Ln^3+^(Ln = Ho, Er, Tm)/Yb^3+^ UCLNPs presented better performances in dual-wavelength synergy, thermosensitivity, emission color tuning, and bioimaging.

## Graphical abstract

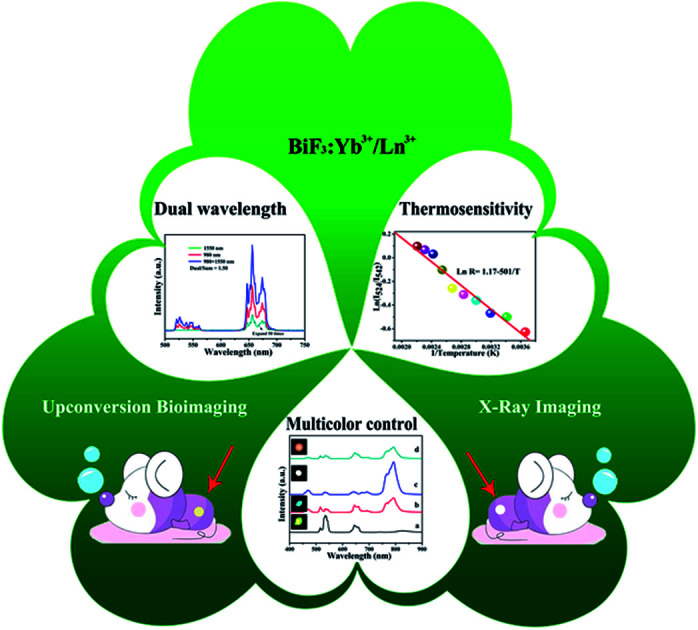

## 1. Introduction

Recently, UCNPs doped with lanthanides (Ln) have made considerable progress.^1–7^ Compared to organic dyes and conventional quantum dot materials, Ln^3+^-doped UCNPs are better in terms of chemical stability, toxicity, optical stability, luminous life, and emission peak, resulting in UCNPs having the advantages of weak auto-fluorescence, low radiation damage, and deep tissue penetration.^8–16^ Therefore, they have great potential in different fields.^17^ It is well known that UC emission can be accurately controlled through adjusting the concentrations of different activator ions (Er^3+^, Tm^3+^, Ho^3+^). Also, white and multicolor UCLNPs have attracted much attention due to their potential applications in backlight sources in color displays and security labeling.^18,19^

It is well known that matrix selecting is an important factor to get desired UCL, in which various host materials, such as sulfide, fluorides, oxygenated compounds, can be used for UC emissions.^20–22^ Among these, fluorides are considered as excellent hosts because of their relatively lower cut-off phonon frequency and lower non-radiative relaxation.^23–27^ Zhang *et al.* reported the excellent matrix NaBiF_4_ without a rare earth ion fabricated by a super facile synthetic method, in which the reaction only took 1 min at room temperature.^28^ Yu *et al.* used the same method to synthesize NaBiF_4_:Yb^3+^/Er^3+^ NPs and BiF_3_:Yb^3+^/Er^3+^ NPs and investigated their UCL properties and temperature sensitivity.^29,30^ However, investigations on multicolor UCL, UCL excited by dual-wavelength, and the bio-imaging of BiF_3_:Yb^3+^/Er^3+^ have not been performed yet, but are very important for their application.^5,18,31^ Herein, this motivated us to further study these excellent matrix BiF_3_:Yb^3+^/Ln^3+^ (Ln = Ho, Er, Tm) NPs in a deeper investigation.

In this work, the cubic phase of BiF_3_:Ln^3+^ (Ln = Ho, Er, Tm)/Yb^3+^ UCNPs was designed and synthesized by the same facile strategy. Their UCL properties were investigated by excitation at 1550 nm and 980 nm, simultaneously. The growth mechanism and the effect of time on their morphology was also explored. The function of different doped concentrations of Er^3+^, Ho^3+^, and Tm^3+^ ions for the multicolor and white emission were also investigated. *In vivo* and X-ray imaging of the BiF_3_:2%Er^3+^/20%Yb^3+^ NPs were studied.

## 2. Experimental

### 2.1 Preparation

All reagents were purchased from Aladdin Chemical Reagent Factory (China) in this research and used without further treatment. The synthesis of the cubic BiF_3_:Ln^3+^/Yb^3+^ UCNPs proceeded as reported in ref. 30 (see ESI†).

### 2.2 Characterization

Details on the instruments used are presented in the ESI.†

### 2.3 *In vitro* cytotoxicity evaluation, and *in vivo* and X-ray imaging

The relevant evaluation^32^ of cytotoxicity *in vitro* and *in vivo* and X-ray imaging were presented in ESI.†

## 3. Results and discussion

### 3.1 Characterization of the phase and morphology

**Fig. 1:**
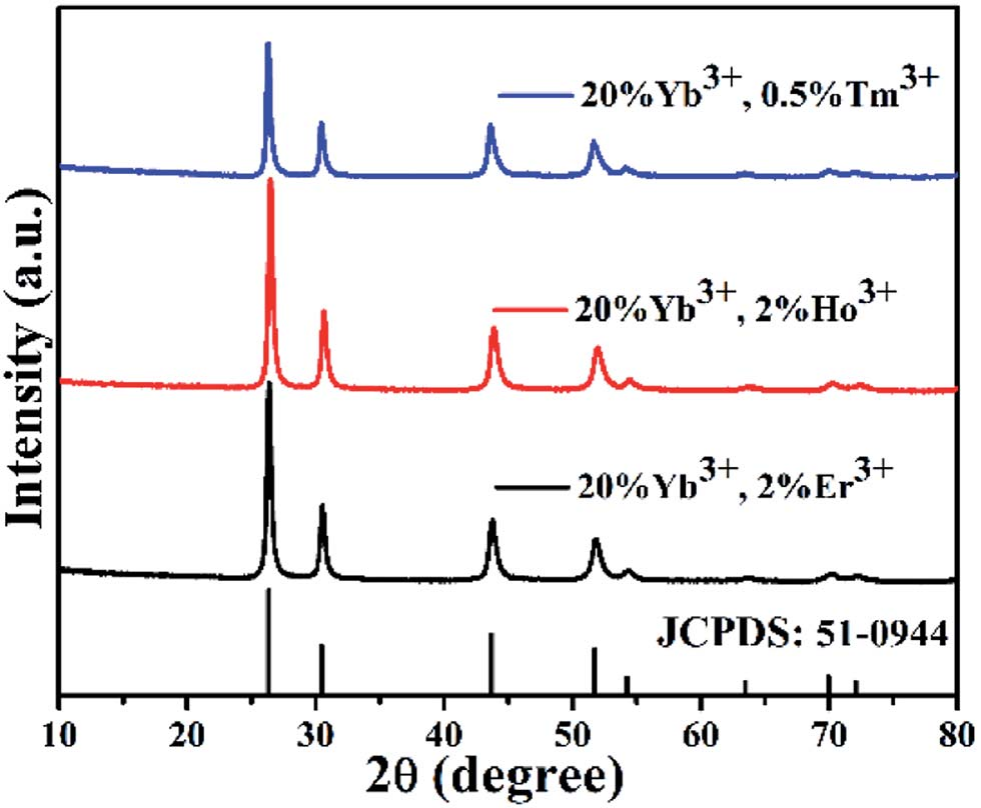
XRD patterns of BiF_3_:Ln^3+^/Yb^3+^.

**Fig. 2:**
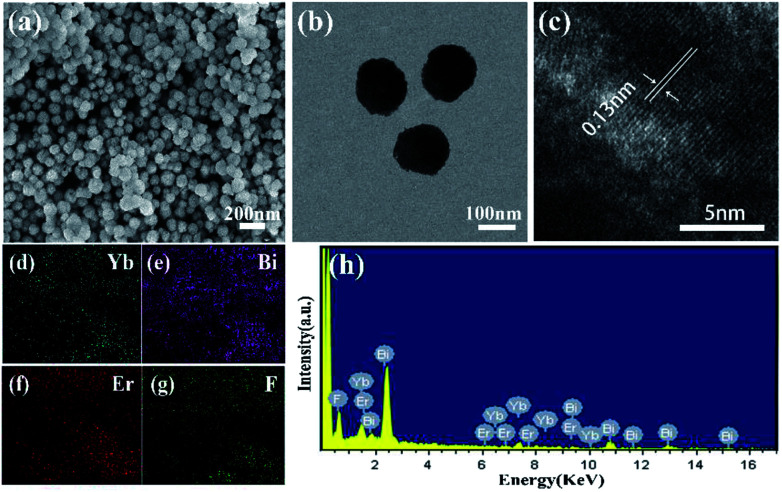
Characterization of BiF_3_:2%Er^3+^/20%Yb^3+^ NPs: (a) SEM image, (b) TEM image, (c) HRTEM image, (d–g) elemental mappings of Yb, Bi, Er, and F, (h) energy-dispersive X-ray (EDX) spectrum.

The crystal phases of BiF_3_:Ln^3+^/Yb^3+^ NPs were determined by XRD as shown in Fig. 1, in which they presented similar crystal cubic phases (JCPDS: 51-0944). The SEM image of BiF_3_:2%Er^3+^/20%Yb^3+^ NPs, as an example, exhibited regular NPs (Fig. 2a), which was confirmed by the TEM image (Fig. 2b). Fig. 2c shows that the *d*-spacing was 0.13 nm, which matched with the crystal plane (331) of the cubic phase BiF_3_ NPs. According to the results of element mapping (Fig. 2d–g), it was clear that the elements of Bi, Yb, Er, and F were uniform on the NPs. These elements were also confirmed by the EDX spectrum (Fig. 2h). BiF_3_:20%Yb^3+^,0.5%Tm^3+^/2% Ho^3+^ are presented in Fig. S1† and their SEM images are shown in Fig. S2,† which further illustrates the formation of BiF_3_:Ln^3+^(Ln = Ho, Er, Tm)/Yb^3+^ UCNPs.

The XPS of BiF_3_:2%Er^3+^/20%Yb^3+^ NPs was measured (Fig. S3a†). Two peaks centered at 159.9 eV and 165.1 eV were assigned to Bi 4f_7/2_ and Bi 4f_5/2_ (Fig. S3b†). The peaks located at 684.4, 165.3, and 186.1 eV were attributed to the BiF_3_:2%Er^3+^/20%Yb^3+^ NPs of Yb 4d, F 1s, and Er 4d, respectively (Fig. S3c–e†).

The stability of the BiF_3_:2%Er^3+^/20%Yb^3+^ NPs was investigated. The TG-DSC curve (see Fig. S4†) showed that the NPs had a mild loss of weight below 600 °C. The loss of water or crystalline water could be calculated at about 100 °C. When heated at 200 °C, the glycol on the surface of the sample gradually disappeared, and the surface defects of the sample gradually decreased. When the temperature reached up to 600 °C, the sample itself began to decompose and then the mass declined linearly.

**Fig. 3:**
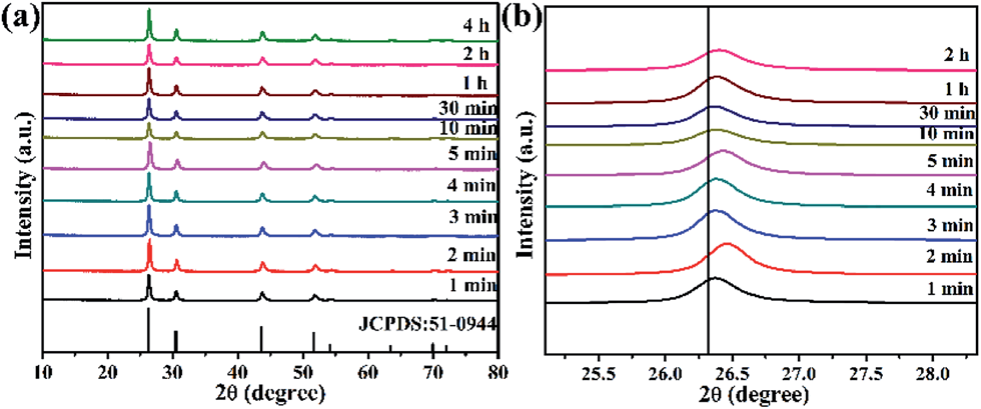
(a) XRD patterns of BiF_3_:2%Er^3+^/20%Yb^3+^ NPs obtained at different reaction times, (b) shifts of the main diffraction peaks of BiF_3_:2%Er^3+^/20%Yb^3+^ NPs.

**Fig. 4:**
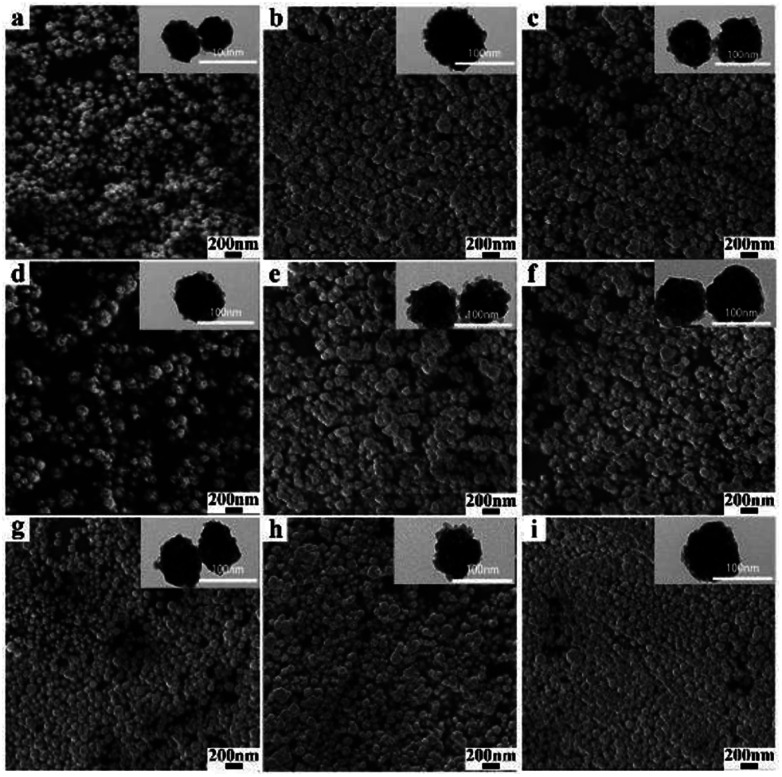
SEM and TEM images of BiF_3_:2%Er^3+^/20%Yb^3+^ NPs obtained at different reaction times: (a) 2 min, (b) 3 min, (c) 4 min, (d) 5 min, (e) 10 min, (f) 30 min, (g) 1 h, (h) 2 h, and (i) 4 h.

To verify whether the reaction time affected the phase and morphology, XRD patterns and SEM/TEM images of BiF_3_:2%Er^3+^/20%Yb^3+^ NPs prepared at different reaction times were investigated (Fig. 3 and 4). The results showed that the diffraction peaks matched with BiF_3_ (JCPDS: 51-0944), indicating that the prepared compounds were single phase and had better crystallinity (Fig. 3). It can be seen from Fig. 3b that the diffraction peaks at 26.34° of Yb^3+^, Er^3+^-doped BiF_3_ had a slight shift compared to BiF_3_ (JCPDS: 51-0944). The reason should be that the ionic radii of both Er^3+^ (1.004 Å) and Yb^3+^ (0.985 Å) are smaller than that of Bi^3+^ (1.17 Å). In the BiF_3_ host lattice, the ions (Yb^3+^, Er^3+^) could enter the BiF_3_ crystal site through substituting for the Bi^3+^ ions or in the interstitial sites or could coexist in these two ways. Different doping methods could make the XRD peaks of the samples prepared at different time shift by different degrees. This indicated that Yb^3+^ and Er^3+^ had been doped into the BiF_3_ matrix.^33^ The XRD of BiF_3_:20%Yb^3+^,0.5%Tm^3+^/2%Ho^3+^ showed similar results, as shown in Fig. S5.†

The SEM and TEM images of BiF_3_:2%Er^3+^/20%Yb^3+^ obtained at different reaction times are presented in Fig. 4. It can be clearly observed that there are a large number of well-dispersed NPs with similar morphologies, indicating that the morphologies were almost unchanged even as the time was prolonged, which was confirmed by the TEM images (insets of Fig. 4). In addition, BiF_3_:20%Yb^3+^,2%Ho^3+^/0.5%Tm^3+^ in Fig. S6 and S7† showed similar results.

**Fig. 5:**
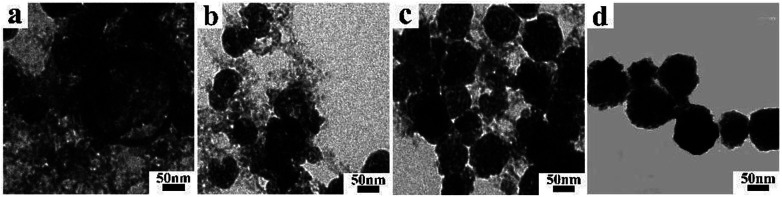
TEM images of BiF_3_:2%Er^3+^/20%Yb^3+^ UCNPs obtained at different reaction times: (a) 5 s, (b) 30 s, (c) 50 s, and (d) 1 min.

**Scheme 1:**
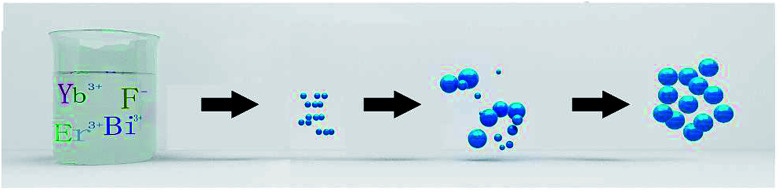
Schematic of the growth mechanism of BiF_3_:2%Er^3+^/20%Yb^3+^.

In order to better understand the formation process of BiF_3_:2%Er^3+^/20%Yb^3+^ within 1 min, the growth mechanism was carefully studied by XRD and TEM at different reaction times (5, 30, 50, and 60 s), as shown in Fig. S8† and 5. Some impurity peaks emerged compared with the standard peaks in 5 s (Fig. S8†) and some small particles appeared in the TEM (Fig. 5a), indicating that the crystal nucleus was completed quickly. As the reaction time was further increased, the impurity peaks gradually disappeared (Fig. S8†) and the basic BiF_3_ spheroids gradually further grew or aggregated (see Fig. 5b and c). These could be deemed as indicating the ripening process of BiF_3_ NPs, meaning that larger spherical NPs were gradually formed due to the thermodynamic minimization of the surface energies of smaller particles, which is commonly known as Ostwald ripening.^34^ BiF_3_:2%Er^3+^/20%Yb^3+^ with regular morphologies were formed and the whole system achieved equilibrium when the time was 1 min (Fig. 5d). Meanwhile, XRD patterns were well matched with the pure cubic phase of BiF_3_ (Fig. S8†). Based on the morphology evolution, the possible growth mechanism is proposed as follows (Scheme 1). First, the precursor was converted to BiF_3_:2%Er^3+^/20%Yb^3+^ nuclei in the nucleation stage. Subsequently, the nuclei grew to rudimental NPs and then underwent the Ostwald ripening process until well-crystallized BiF_3_ NPs were formed, therefore the process could be described as “Nucleation → Crystallization → Ostwald ripening” (Scheme 1).

**Fig. 6:**
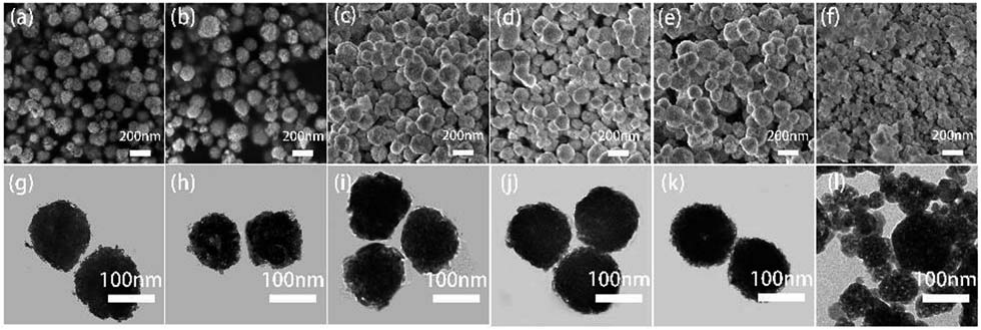
SEM and TEM images of BiF_3_:20%Yb^3+^/2%Er^3+^ NPs obtained at different temperatures: (a and g) −25 °C, (b and h) 0 °C, (c and i) 30 °C, (d and j) 50 °C, (e and k) 70 °C, and (f and l) 100 °C.

The influence of the temperature on the phase and morphology of BiF_3_:2%Er^3+^/20%Yb^3+^ were also investigated by XRD, SEM, and TEM (Fig. S9† and 6). It could be seen that the XRD spectra of NPs obtained at different temperatures (−25, 0, 30, 50, and 70 °C) matched well with the standard cubic phase (Fig. S9†). No impurities peaks were found in these patterns, indicating these samples had higher purity. However, a few new diffraction peaks appeared at 100 °C, indicating that the pure cubic phase of BiF_3_:2%Er^3+^/20%Yb^3+^ NPs tended to be formed at a lower temperature. Meanwhile, SEM images of BiF_3_:2%Er^3+^/20%Yb^3+^ NPs synthesized at different temperatures, sizes, and morphologies also showed no obvious changes, except that they became irregular at 100 °C (Fig. S9† and 6f). The corresponding TEM images are shown in Fig. 6g–l, in which the results were in good agreement with the XRD results. From the above analysis, it could be concluded that the regular BiF_3_:2%Er^3+^/20%Yb^3+^ NPs could be formed at lower temperature under co-precipitation conditions.

### 3.2 UCL of BiF_3_:Ln^3+^(Ln = Ho, Er, Tm)/Yb^3+^

#### 3.2.1 Effect of calcination temperature on the stability of UCL

**Fig. 7:**
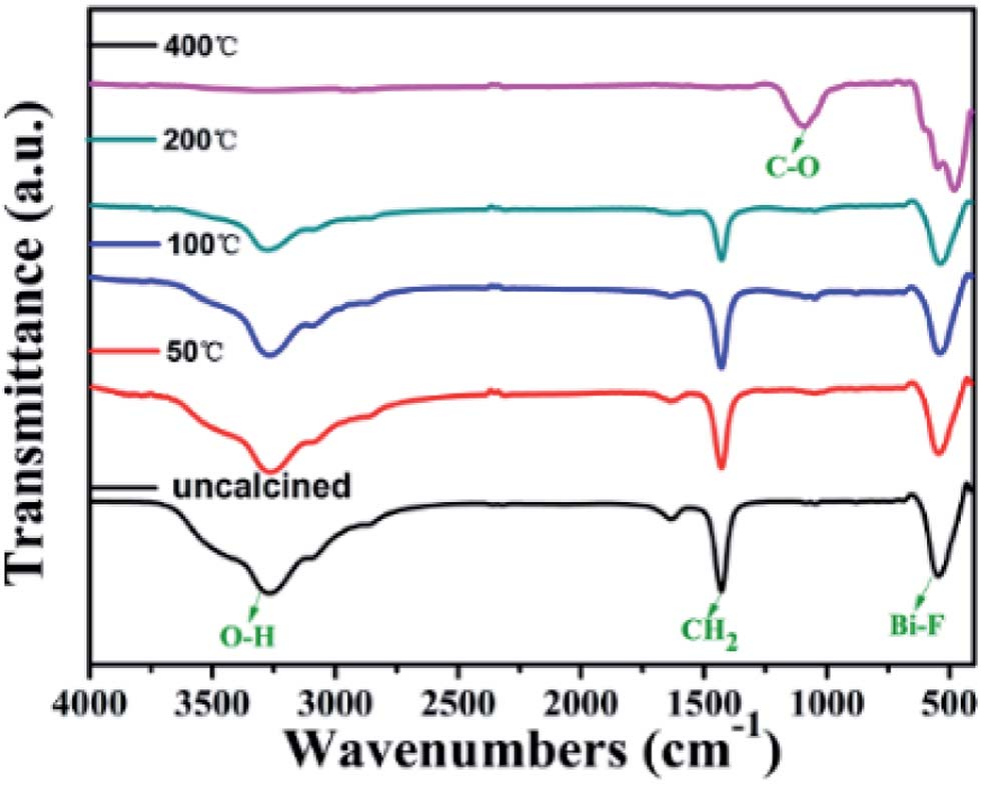
FT-IR spectra of BiF_3_:2%Er^3+^/20%Yb^3+^ calcinated at different temperatures.

Fig. S10† presents the UCL spectra of BiF_3_:2%Er^3+^/20%Yb^3+^ excited at 980 nm for different times. It can be seen that when prepared without calcination, its emission intensity gradually increased with prolonging the irradiation time (Fig. S10a†) and reached the maximal value in 7 min. Similarly, the variation trend of the UCL intensity of BiF_3_:2%Er^3+^/20%Yb^3+^ annealed at 50 °C was the same as that without calcination (Fig. S10b†). However, the UCL intensity did not change with increasing irradiation time when it was annealed at 100 °C, 200 °C, or 400 °C (Fig. S10c–e†). The reason for this might be that there was a large amount of hydroxyl groups (OH) on the sample's surface before calcination or certain temperature, which could increase the non-radiative relaxation rate and decrease the luminescence efficiency.^35^ The results could be further confirmed by FT-IR and XRD studies of BiF_3_:2%Er^3+^/20%Yb^3+^ before and after calcination. The XRD patterns and FT-IR spectra of BiF_3_:2%Er^3+^/20%Yb^3+^ NPs at different calcination temperatures were recorded, as shown in Fig. S11† and 7. The diffraction peaks were matched with a cubic phase of BiF_3_, except when annealed at 400 °C in Fig. S11.† The reason might be that the temperature reached its phase inversion temperature and then the crystalline phase began to change. The morphologies of samples calcined at different temperatures were also analyzed, as shown in Fig. S12.† From the figure, we can clearly see that the morphology of calcined samples became irregular when the calcination temperature was 400 °C. This result was consistent with the XRD results. According to the FT-IR spectra measured for the calcinated NPs (Fig. 7), the intensity of the characteristic peaks changed. The band at 552 cm^−1^ arose from the vibration mode of Bi–F, which showed almost no change, but then changed at 400 °C to form another phase. The band approximately at 3388 cm^−1^ and 1650 cm^−1^ ascribed to O–H and some organic molecular decreased with the rising calcination temperature. The enhancement of UCL intensity might be caused by the reduction in the number of OH groups^35^ and in the organic component. This was in agreement with the result of TG-DSC. BiF_3_:2%Er^3+^/20%Yb^3+^ treated at 200 °C was used for the following investigation on UCL.

#### 3.2.2 Investigation on UCL mechanism

**Fig. 8:**
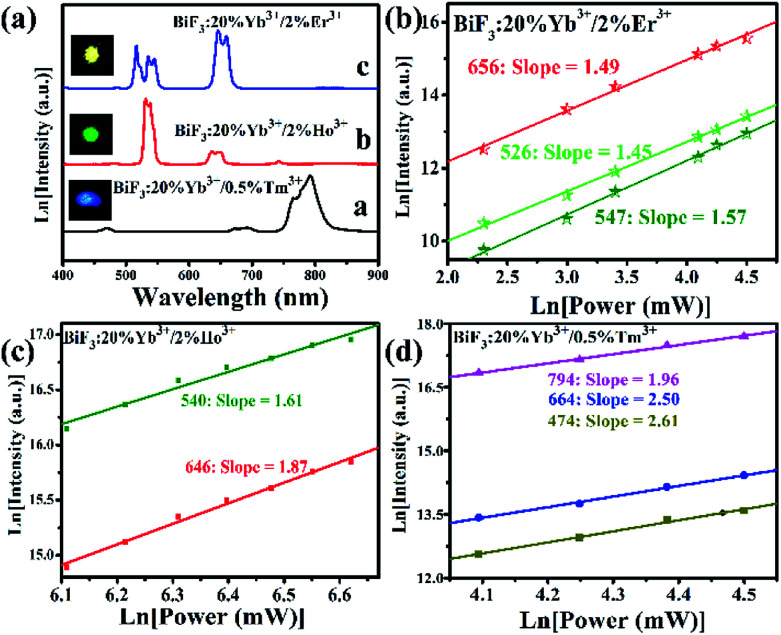
(a) UC emission spectra and corresponding luminescence photographs, (b–d) pump power dependence of blue, green, red, and NIR UCL emission intensities in BiF_3_:Ln^3+^ NPs excited at 980 nm.

The UC emission spectra of BiF_3_:Ln^3+^ (Ln = Tm, Er, Ho)/Yb^3+^ NPs irradiated at 980 nm were measured and the corresponding results are given in Fig. 8a. It could be seen that there were three different colors. To better understand the mechanism of every UCL, the variation of UCL intensities with pump power density was measured (Fig. 8b–d). It is well known that the relationship between the pump power density (*P*) and luminescence intensity (*I*) is *I* = *P*^*n*^, by which information of the *n* photons in the UCL process can be provided.^36^ As presented in Fig. 8b, the slopes for the 526, 547, and 656 nm bands were 1.45, 1.57, and 1.49, respectively, showing a two-photon absorption process for the BiF_3_:2%Er^3+^/20%Yb^3+^ NPs. As presented in Fig. 8c, the slopes were 1.87 and 1.61 for Ho^3+^ ion, meaning a two-photon process for BiF_3_:2%Ho^3+^/20%Yb^3+^ NPs. The slopes for BiF_3_:0.5%Tm^3+^/20%Yb^3+^ NPs were 1.97, 2.50, and 2.60, corresponding to two-photon (red NIR) and three photon (red and blue) energy-transfer processes in Fig. 8d.

The possible UCL mechanism of Yb^3+^, Er^3+^, Ho^3+^, and Tm^3+^ ions are shown in Fig. S13.† For BiF_3_:2%Er^3+^/20%Yb^3+^ NPs, the Yb^3+^ ion absorbs a photon and is excited to ^2^F_5/2_, then drops back to the ground state and transfers the energy to a nearby Er^3+^ ion to populate the ^4^I_11/2_. The Yb^3+^ can then transfer the second photon to populate the ^4^F_7/2_ and relax to ^2^H_11/2_ or ^4^S_3/2_ levels non-radiatively. Through non-radiative relaxation, the ^4^I_11/2_ level can also relax to the ^4^I_13/2_ level and after that a transferred photon from Yb^3+^ ions can populate the ^4^F_9/2_. Red emission is transmitted through ^4^F_9/2_ → ^4^I_15/2_ of Er^3+^, as shown in Fig. S13a.† The red and blue emissions of BiF_3_:20%Yb^3+^/0.5%Tm^3+^ NPs could be confirmed as a three-photon process, as shown in Fig. S13b.† The ^1^G_4_ level could be populated by a three-step energy transfer of Yb^3+^ through ^3^H_4_ and ^3^F_4_. From the transitions of ^1^G_4_ → ^3^F_4_, ^3^H_4_ → ^3^H_6_ and ^1^G_4_ → ^3^H_6_, red, NIR, and blue emissions of Tm^3+^ could be obtained, respectively. As for BiF_3_:20%Yb^3+^/2%Ho^3+^ NPs, red emission through the bridge of the ^5^I_7_ level was pumped to the ^5^F_5_ level and the green emission was then a two-photon process (Fig. S13b†).^18^

#### 3.2.3 Multicolor tuning and bright white emission

**Fig. 9:**
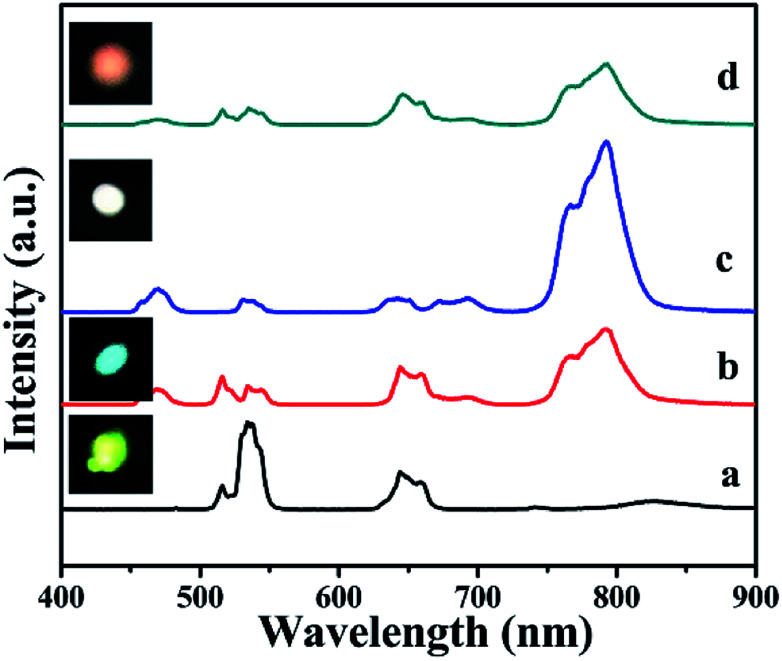
UCL emissive spectra and corresponding luminescence photographs of: (a) BiF_3_:20%Yb^3+^/1%Er^3+^/0.15%Ho^3+^, (b) BiF_3_:20%Yb^3+^/1%Er^3+^/0.5%Tm^3+^, (c) BiF_3_:20%Yb^3+^/0.5%Tm^3+^/0.15%Ho^3+^, (d) BiF_3_:20%Yb^3+^/1%Er^3+^/0.5%Tm^3+^/0.15%Ho^3+^ NPs excited at 980 nm.

In order to expand those UCNPs potential applications, the bright white and multicolor UCL has been studied. To get multicolor emission, such as bright white emission, the concentrations of Er^3+^, Ho^3+^, and Tm^3+^ ions could be properly adjusted, in which different samples were obtained (Table S1†). The bright yellow green, pinkish, bright white, and bluish white UCL emissive spectra and photographs are shown in Fig. 9, which agreed well with the results of the color coordinate in Fig. S14.† Those results indicated that BiF_3_ nanomaterials have potential applications as backlight sources in color displays and security labeling.

#### 3.2.4 Performance of UCL under dual-wavelength excitation (1550 nm and 980 nm)

**Fig. 10:**
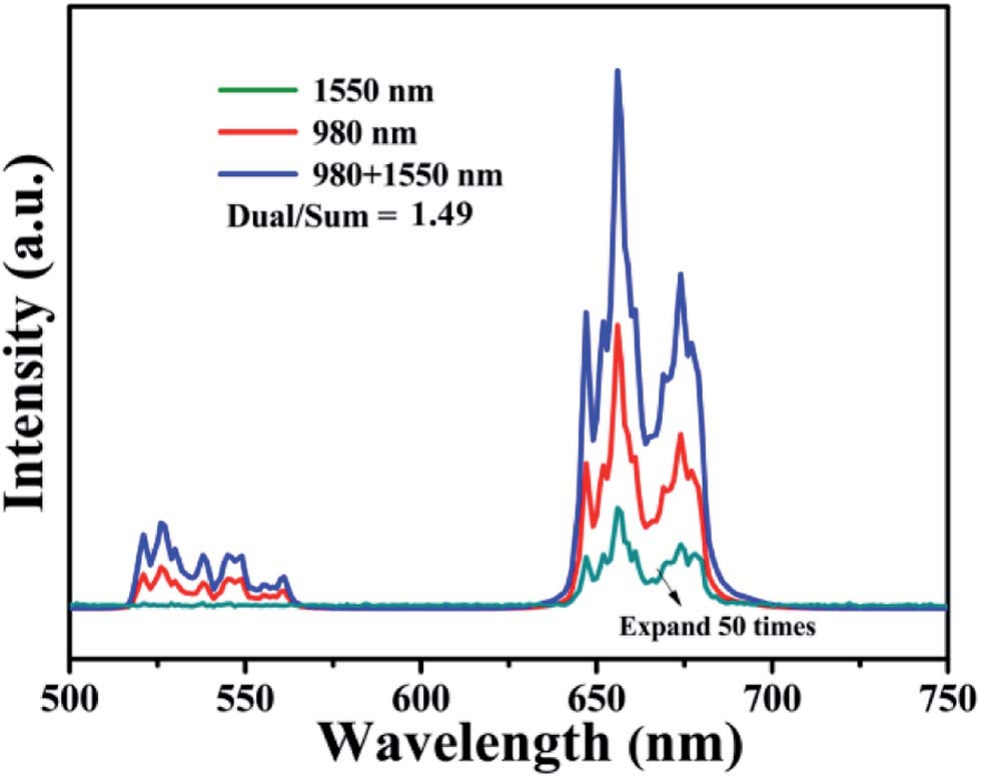
UC emission spectra of BiF_3_:2%Er^3+^/20%Yb^3+^ under 980 nm, 1550 nm, or simultaneous dual-wavelength (1550 nm and 980 nm) excitation (*P*_1550_ = *P*_980_ = 100 mW).

It was necessary to investigate the UC process with dual-wavelength excitation, which is considered as potentially a convenient way to improve the efficiency of power conversion in next-generation solar cells.^37–39^ According to Fig. 10, the ratio of green/red emission intensities was similar. Under dual-excitation at 1550 nm and 980 nm, the intensity of UCL was greater than the sum of the intensities at 1550 nm and 980 nm, respectively. It was 1.49 times the sum of the separate single wavelength excitations, indicating there was a dual-wavelength synergistic effect in this system.^40,41^

#### 3.2.5 Thermometric property of BiF_3_:2%Er^3+^/20%Yb^3+^ NPs

It was well known that the change of the transitions of ^2^H_11/2_ → ^4^I_15/2_ (524 nm) and ^4^S_3/2_ → ^4^I_15/2_ (542 nm) at different temperatures owing to ^2^H_11/2_ and ^4^S_3/2_ levels were related to the thermal coupling of Er^3+^ ion. The ratio (*R*) of emission intensities at 524 nm and 542 nm followed the formula based on the Boltzmann distribution theory:^42–44^1
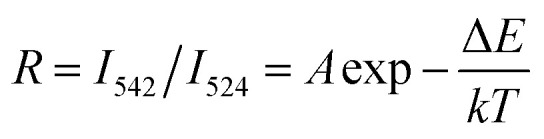
where *A* is the proportionality constant, Δ*E* is the energy gap between ^2^H_11/2_ and ^4^S_3/2_ levels, *T* is the absolute temperature, and *k* is the Boltzmann's constant. Eqn (1) could also be expressed as:2
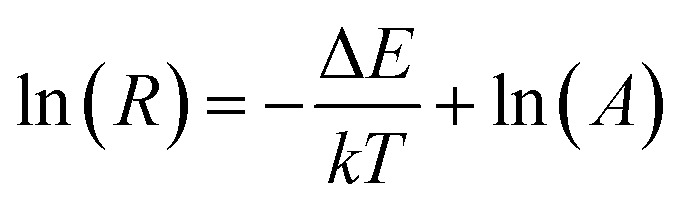


**Fig. 11:**
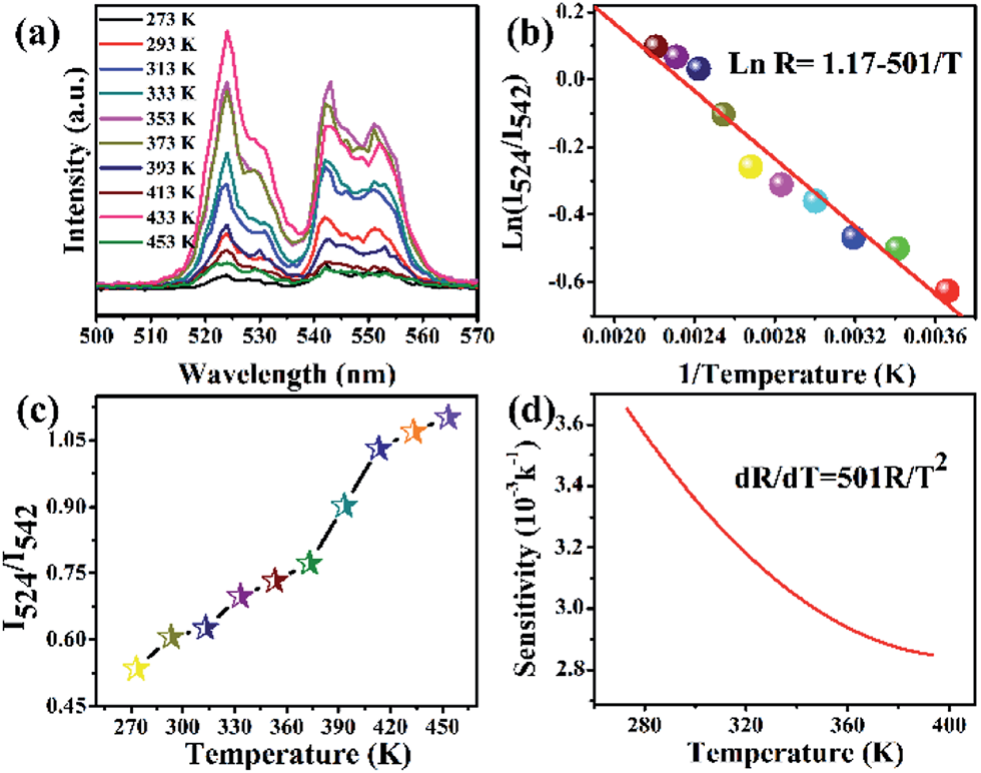
(a) Green UC emission spectra of BiF_3_:2%Er^3+^/20%Yb^3+^ UCNPs in the temperature range of 273–453 K, (b) plot of ln(*R*) *versus* 1/*T*. (c) *R* values of green emissions intensities at different temperatures, (d) relative sensitivity.

Fig. 11a presents the UC emission spectra of BiF_3_:2%Er^3+^/20%Yb^3+^ excited at 980 nm with various temperatures. For green (524 nm and 542 nm) emissions, the UC emission intensities changed with increasing temperature. Fig. 11b presents a plot of 1/*T versus* ln(*R*), in which the slope (−Δ*E*/*k*) is equal to −501 and the intercept is 1.17. It could be also seen from Fig. 11c that *R* increased with the increasing temperature and presented a certain rule. In addition, the sensor sensitivity is another very important factor. The sensor sensitivity (*S*) can be calculated by using the following formula:^45–48^3
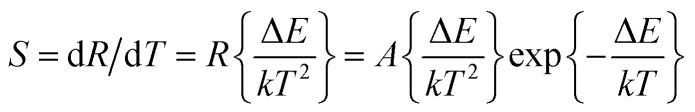


It was noteworthy that the sensitivity exhibited a downward tendency with the increasing temperature from 273 K to 453 K and its max value was about 0.0036 K^−1^ at 273 K (Fig. 11d), suggesting that the BiF_3_:2%Er^3+^/20%Yb^3+^ NPs had a certain thermometric property, which might be employed as a temperature sensor.

#### 3.2.6 Cell toxicity test

**Fig. 12:**
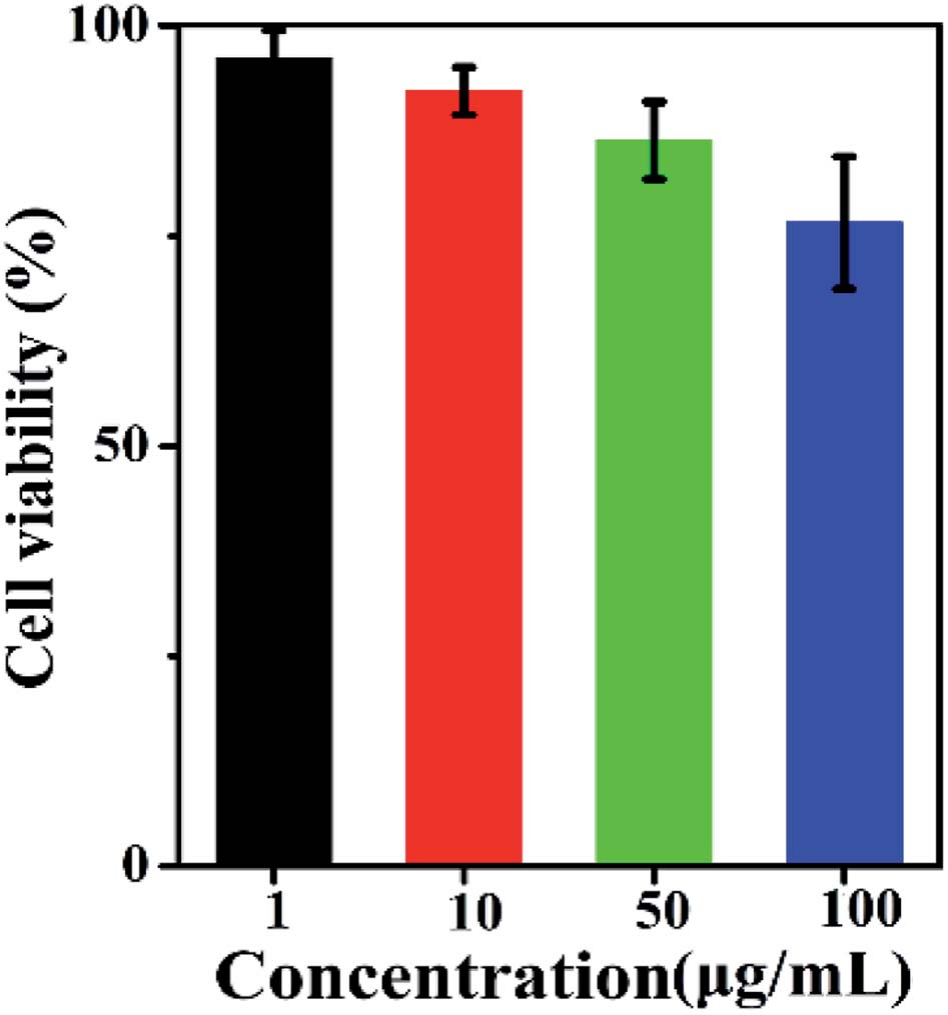
Cell viability of B16-F10 cells incubated with different concentrations of BiF_3_:2%Er^3+^/20%Yb^3+^ phospholipid aqueous solution.

In order to evaluate the potential biosafety, cytotoxicity tests must be investigated.^49,50^ As illustrated in Fig. 12, the cytotoxicity of a NPs phospholipid aqueous solution with different concentrations was assessed by CCK-8 assay under 5% CO_2_ at 37 °C for 24 h. The viability of the B16-F10 cells decreased to 90% at a density of 1 μg mL^−1^. When the concentration varied from 50 μg mL^−1^ to 100 μg mL^−1^, the cellular viability of B16-F10 cells decreased to 82%, indicating that the UCNPs phospholipid aqueous solution presented lower cytotoxicity.

#### 3.2.7 *In vivo* imaging

**Fig. 13:**
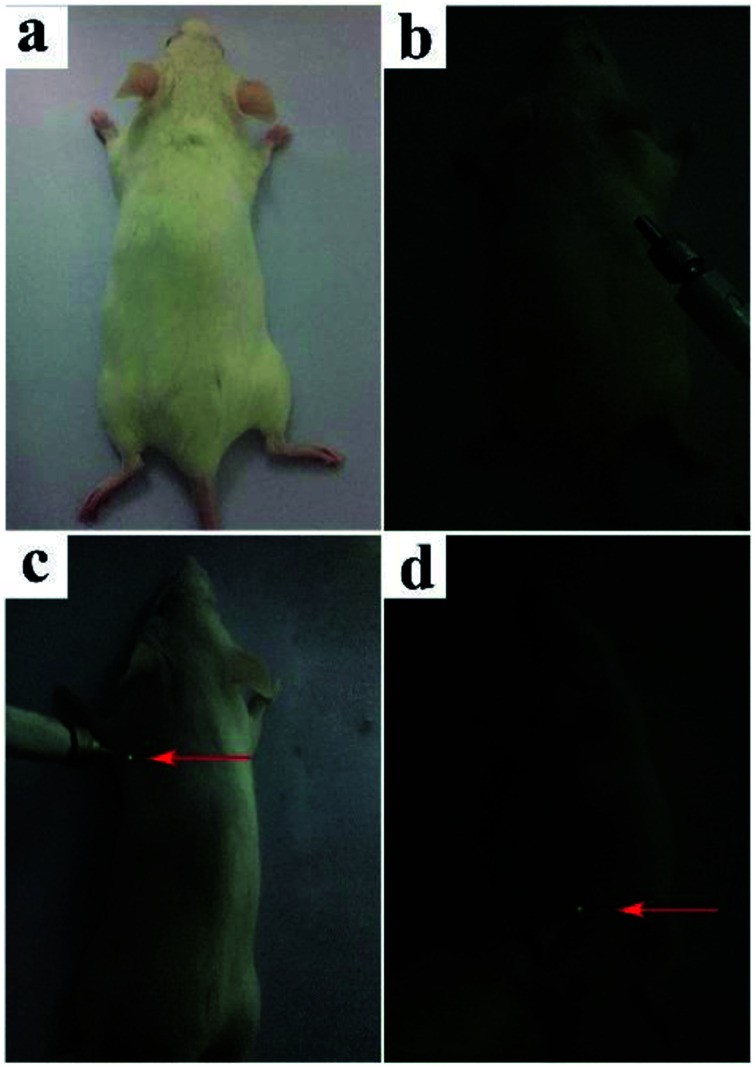
*In vivo* UC imaging of a Kunming mouse: (a and b) without injection of UCNPs, (c) subcutaneous injection, and (d) intraperitoneal injection of UCNPs [100 μL (100 μg mL^−1^) for c and d].

100 μL BiF_3_:2%Er^3+^/20%Yb^3+^ UCNPs phospholipid aqueous solution (100 μg mL^−1^) was injected into a Kunming mouse subcutaneously or intraperitoneally for deeper tissue imaging. In a comparative experiment, the luminous point on a Kunming mouse without treatment with NPs by exciting at 980 nm could not be observed with UCL (Fig. 13a and b). On the other hand, after intraperitoneal and subcutaneous injection in the corresponding area (100 μL, 100 μg mL^−1^), yellow UCL could be observed in dark upon the excitation at 980 nm laser (Fig. 13c and d). These indicated that UCNPs had a high contrast and deep penetration for *in vivo* optical bioimaging, which could be used as probe.^5^

#### 3.2.8 *In vivo* X-ray imaging

**Fig. 14:**
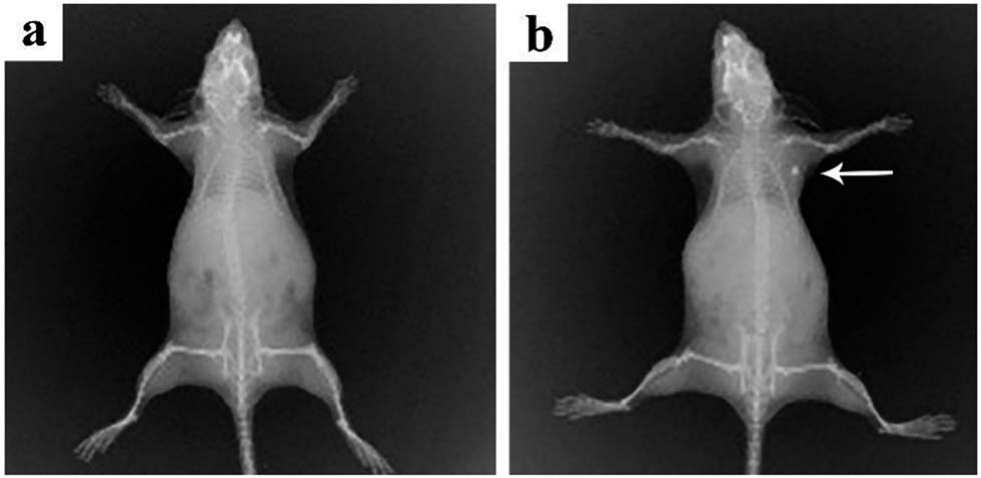
*In vivo* X-ray imaging of a Kunming mouse before (a) and after (b) subcutaneous injection of 200 μL of BiF_3_:2%Er^3+^/20%Yb^3+^ NPs.

A certain amount of BiF_3_:2%Er^3+^/20%Yb^3+^ (200 μL, 100 μg mL^−1^) UCNPs phospholipid aqueous solution was injected subcutaneously in a Kunming mouse for X-ray imaging. Obvious X-ray signals marked by a white arrow could be observed for the Kunming mouse injected with NPs (Fig. 14b) compared to non-injection (Fig. 14a). It has been proven that they can absorb X-rays. The results showed that they could be utilized as X-ray imaging contrast agents.

## 4. Conclusions

In summary, multi-functional BiF_3_ UCNPs were successfully synthesized by a facile co-precipitation method, both below and above room temperature (−25 °C, 100 °C). The growth mechanism indicated that the obtained BiF_3_ NPs were formed through the growth process of “Nucleation → Crystallization → Ostwald ripening”. Fluorescence intensity ratio technology was used to investigate the temperature sensitivity of BiF_3_:2%Er^3+^/20%Yb^3+^ and the max sensitivity was found to be 0.0036 K^−1^ at 273 K. The integrated emission of BiF_3_:2%Er^3+^/20%Yb^3+^ NPs excited by dual-wavelength simultaneously was 1.49 times more than that excited by two single wavelengths (1550 nm and 980 nm), respectively. Multicolor and bright white light could be obtained upon excitation at 980 nm by modulating the content of Ho^3+^, Er^3+^, and Tm^3+^, by which they might be used as security labeling and backlight sources for color displays. The low cytotoxicity of BiF_3_:2%Er^3+^/20%Yb^3+^ was confirmed by cell toxicity tests with phospholipid aqueous solution. BiF_3_:2%Er^3+^/20%Yb^3+^ NPs presented better properties of penetration and contrast in the *in vivo* imaging and X-ray imaging tests, indicating that they could be used as potential probe and contrast agents in *in vivo* optical bioimaging.

## Live subject statement

All animal procedures were performed in accordance with the Guidelines for Care and Use of Laboratory Animals of Zhengzhou University and experiments were approved by the Animal Ethics Committee of Henan Laboratory Animal Center.

## Conflicts of interest

There are no conflicts to declare.

## Supplementary Material

RA-009-C9RA01018A-s001
