# Supplementary material for: Multifunctional BiF3:Ln3+ (Ln = Ho, Er, Tm)/Yb3+ nanoparticles: an investigation on the emission color tuning, thermosensitivity, and bioimaging
Source: RSC Adv. 2019 Apr 8;9(19):10889–96. doi: 10.1039/c9ra01018a (PMC9062528; doi:10.1039/c9ra01018a)
Supplement: RA-009-C9RA01018A-s001 [file RA-009-C9RA01018A-s001.pdf]

Electronic Supplementary Material (ESI) for RSC Advances.
This journal is © The Royal Society of Chemistry 2019

Multifunctional BiF_3:Ln^{3+}(Ln = Ho, Er, Tm)/Yb^{3+} nanoparticles: investigation on the emission colour tuning, thermosensitivity and bio imaging

Xinxin Yan[a], Tiesheng Li[a*], Linna Guo[a], Honglei Li[a], Penglei Chen[b], Minghua Liu[b*]

[a]*College of Chemistry and Molecular Engineering, Zhengzhou University, The Key Lab of Chemical Biology and Organic Chemistry of Henan Province, The Key Lab of Nano-information Materials of Zhengzhou, Zhengzhou, 450001, P. R. China.*

[b]*Beijing National Laboratory for Molecular Science, Institute of Chemistry, Chinese Academy of Sciences, Beijing, 100190, P. R. China.*

*Corresponding author. Tiesheng Li,

E-mail address: lts34@zzu.edu.cn

Tel.: +86-371-67766667 (Office)

Fax.: +86-371-67766667 (Office)

1. Experimental

1.1 Materials

Analytical grade bismuth nitrate pentahydrate (Bi $(NO_3)_3 \cdot 5H_2O$ AR grade), ammonium fluoride (NH_4F, 98%), Yttrium oxide (Yb_2O_3, 99.99%), Erbium oxide (Er_2O_3, 99.99%), Thulium oxide (Tm_2O_3, 99.99%), Holmium oxide (Ho_2O_3, 99.99%), and Ethylene glycol (EG) were purchased from Aladdin Chemical Reagent Factory (China). The corresponding rare earth oxides are dissolved in dilute HNO_3 under stirring and then the solvent is evaporated to obtain all the rare earth nitrates.

1.2 Preparation of cubic BiF_3:Ln^{3+} (Ln = Ho, Er, Tm)/Yb^{3+} UCNPs

0.78 mmol $Bi(NO_3)_3 \cdot 5H_2O$, 0.02 mmol $Er(NO_3)_3$ and 0.20 mmol $Yb(NO_3)_3$ were added to 10 mL EG in a beaker under vigorously stirring. Then 25 ml EG with 5 mmol NH_4F was added into the above solution and stirred for 1 min. Subsequently, the resulting suspension was separated and washed with deionized water and ethanol several times by centrifugation, after that dried at room temperature. In addition, 20%Yb^{3+}, 2%Ho^{3+}/0.5%Tm^{3+} co-doped BiF_3 UCNPs were obtained by the same method. The similar procedure was used for comparative experiments at different temperatures and reaction time.

1.3 Characterization

XRD experiments were carried out with a Bruker D8 VENTURE diffractometer (Bruker, German) using Cu-Ka radiation. SEM images were examined using a Hitachi S-4800 system. TEM images were obtained using FEI TECNAIG2F20-S-TWIN transmission electron microscope. TG-DSC measurements were measured with an SDT

Q600 instrument (heating rate (5°C min^{-1}), 200-1200°C, under an air flow of 200 mL/min). XPS was carried out with a VG Scientific Model ESCALab220i-XL electron spectrometer using 300 W Al Karadiation. Using a HORIBA JOBIN YVON Fluorolog-3 spectrofluorometer system to collect two continuous 980 nm and 1550 nm laser diode. In order to analyze chemical components, FT-IR was used (Nicolet NEXUS 670).

1.4 In vitro cytotoxicity evaluation

To evaluate the cytotoxicity of BiF_3:20%Yb^{3+}/2%Er^{3+} UCNPs, CCK-8 assays were used on B16-F10 cells incubated with UCNPs.B16-F10 cells were seeded in a 96-well plate at a density of 8000 cells per well and cultured in 5% CO_2 at 37°C for 24 h. The BiF_3:20%Yb^{3+}/2%Er^{3+} UCNPs (1 mg/mL) were dispersed in phospholipid aqueous solution (2 mg/mL) by ultrasound for 6 h (1:1).

1.5 In vivo imaging

100 μL of BiF_3:20%Yb^{3+}/2%Er^{3+} NPs phospholipid aqueous solution with concentration of 100 μg/mL was injected intraperitoneally and subcutaneously into a kunming mouse. In order to eliminate the interference of 980 nm excitation laser, a short-pass filter (700 nm) was used.

1.6 In vivo X-Ray imaging

200 μL of BiF_3:20%Yb^{3+}/2%Er^{3+} NPs phospholipid aqueous solution with concentration of 100 μg/mL was subcutaneously injected into the mouse. Then, in vivo X-ray imaging was tested by a BRUKER In Vivo FXPRO imaging system. All animal procedures comply with the care and use regulations of animal institutions.

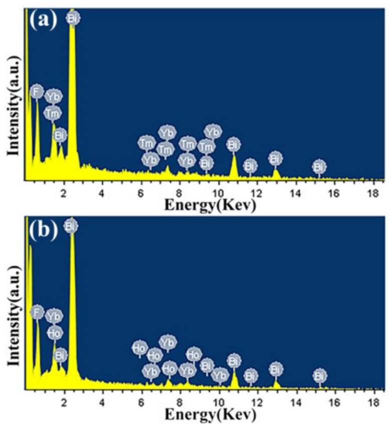

Figure S1 Energy-dispersive X-ray (EDX) spectroscopy: (a) BiF_3:20%Yb^{3+}/0.5%Tm^{3+}, (b) BiF_3:20%Yb^{3+}/2%Ho^{3+}.

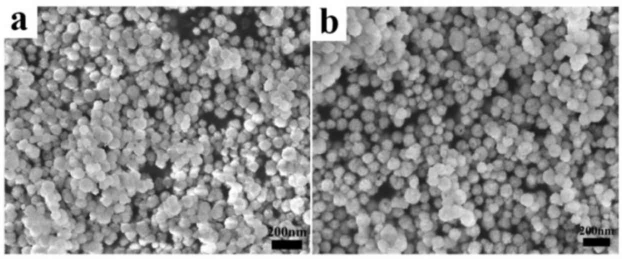

Figure S2 SEM images: (a) BiF_3:20%Yb^{3+}/2%Ho^{3+}, (b) BiF_3:20%Yb^{3+}/0.5%Tm^{3+}. (obtained in 1 min at room temperature)

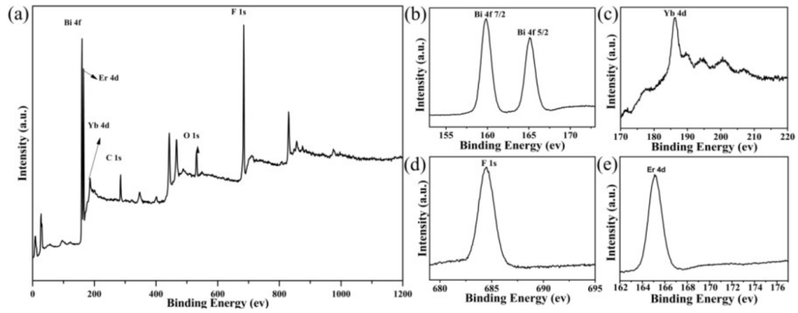

Figure S3 XPS survey spectra of BiF_3:20%Yb^{3+}/2%Er^{3+} NPs. (a) survey, (b) Bi 4f, (c) Yb 4d, (d) F 1s and (e) Er 4d.

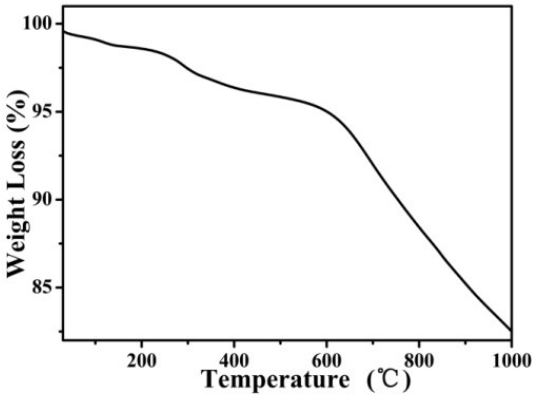

Figure S4 TG-DSC curve of the sample BiF_3:2%Er^{3+}/20%Yb^{3+}.

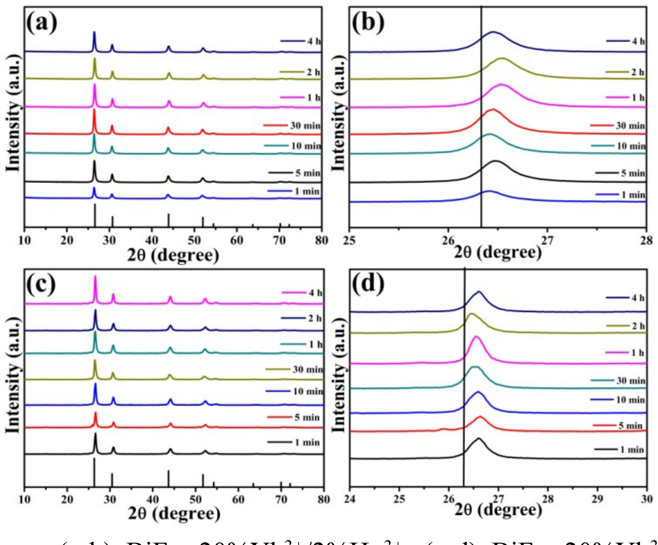

Figure S5 XRD patterns: (a-b) BiF_3: 20%Yb^{3+}/2%Ho^{3+}, (c-d) BiF_3: 20%Yb^{3+}/0.5%Tm^{3+} NPs obtained at different reaction time.

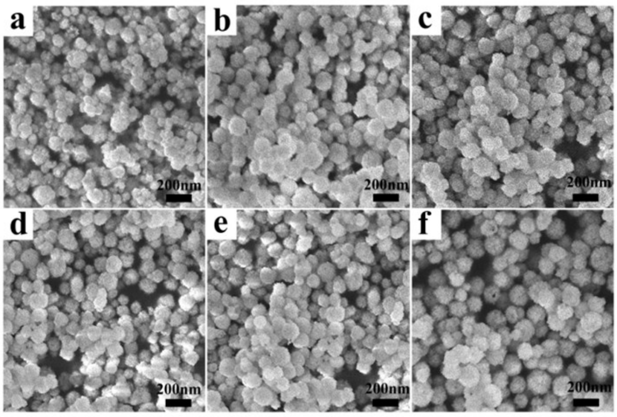

Figure S6 SEM images of BiF_3:20%Yb^{3+}/2%Ho^{3+} NPs obtained at different reaction time. (a) 5 min, (b) 10 min, (c) 30 min, (d) 1 h, (e) 2 h, and (f) 4 h.

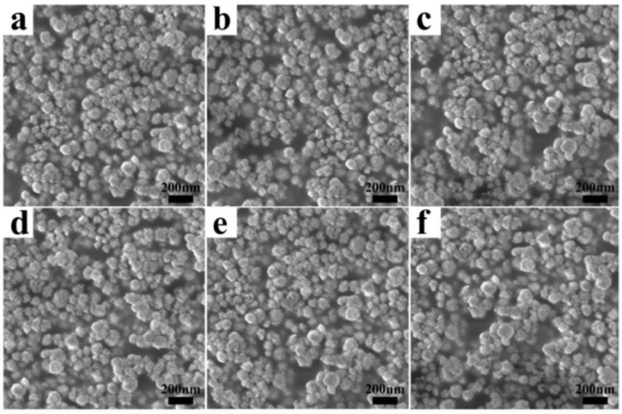

Figure S7 SEM images of BiF_3: 20%Yb^{3+}/0.5%Tm^{3+} NPs obtained at different reaction time. (a) 5 min, (b) 10 min, (c) 30 min, (d) 1 h, (e) 2 h, and (f) 4 h.

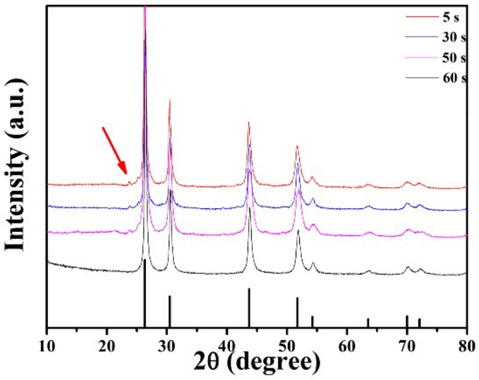

Figure S8 XRD patterns of the products obtained at different reaction time of BiF_3: 20%Yb^{3+}/2%Er^{3+} nanoparticles.

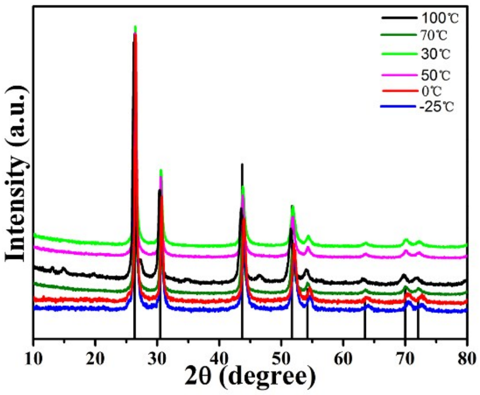

Figure S9 XRD patterns of BiF_3: 20%Yb^{3+}/2%Er^{3+} NPs obtained at different temperatures (T = -25, 0, 30, 50, 70 and 100℃).

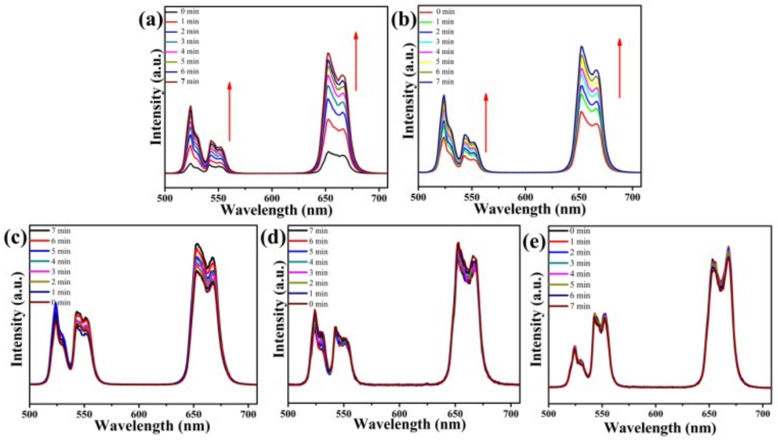

Figure S10 UCL spectra of BiF_3: 20%Yb^{3+}/2%Er^{3+} nanoparticles under excitation of 980 nm with (a) uncalcined; (b) calcined at 50 °C; (c)calcined at 100 °C; (d) calcined at 200 °C and (d) calcined at 400 °C.

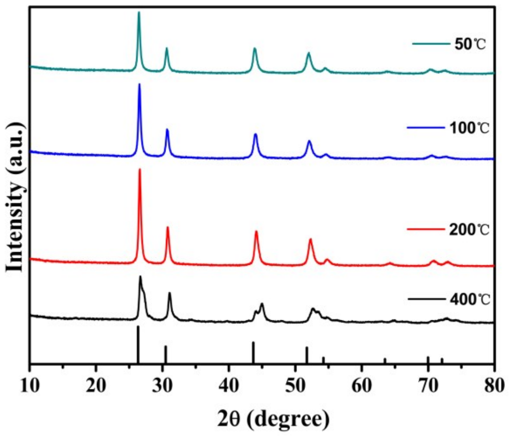

Figure S11 XRD patterns of BiF_3: 20%Yb^{3+}/2%Er^{3+} with different calcination temperatures.

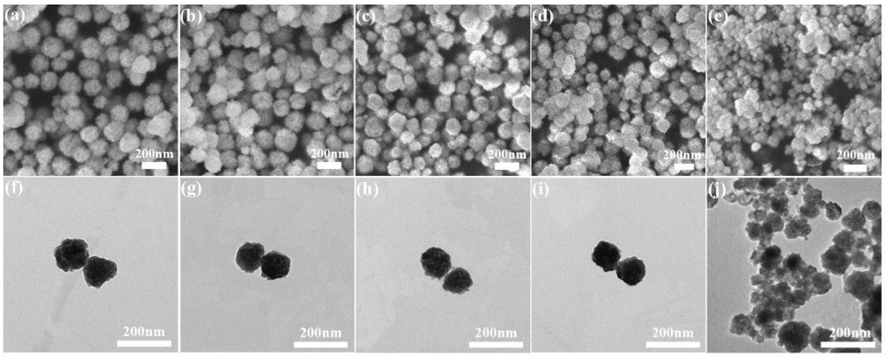

Figure S12 SEM and TEM images of BiF_3:2%Er^{3+}/20%Yb^{3+} NPs obtained at different calcination temperatures. (a.f) uncalcined, (b.h) calcined at 50 °C, (c.i) calcined at 100 °C, (d.j) calcined at 200 °C, and (e.k) calcined at 400 °C.

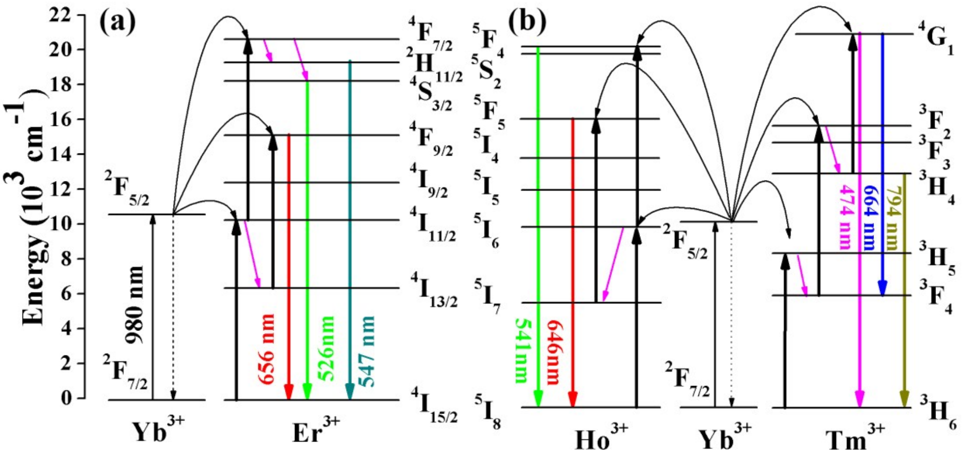

Figure S13 Energy level diagrams of Yb^{3+}, Er^{3+}, Tm^{3+} and Ho^{3+} ions and proposed UC mechanisms in BiF_3:Ln^{3+} NPs.

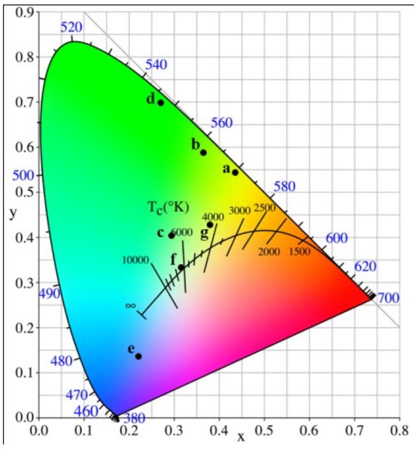

Figure S14 The CIE 1931 chromaticity diagram of BiF_3: Ln^{3+} NPs excited with a 980 nm laser.

Table S1 The summary of chemical composition, chromaticity coordinates (x, y) and emission color of the BiF_3: Ln^{3+} NPs.

Sample	Chromaticity Coordinates (x, y)	Emission color
(a) BiF_3:20％Yb^{3+} /2％Er^{3+}	(0.4355,0.5438)	Yellow
(b) BiF_3: 20％Yb^{3+} /1％Er^{3+}/0.15％Ho^{3+}	(0.3667,0.5897)	Yellow green
(c)BiF_3: 20％Yb^{3+} /1％Er^{3+}/0.5％Tm^{3+}	(0.2947,0.4059)	Bluish white
(d) BiF_3: 20％Yb^{3+} /2％Ho^{3+}	(0.269,0.6978)	Green
(e) BiF_3: 20％Yb^{3+} /0.5％Tm^{3+}	(0.2225,0.1364)	Purplish blue
(f) BiF_3: 20％Yb^{3+} /0.5％Tm^{3+}/0.15％Ho^{3+}	(0.3166,0.3327)	White
(g) BiF_3: 20％Yb^{3+} /1％Er^{3+}/0. 5％Tm^{3+}/0.15％Ho^{3+}	(0.3783,0.4264)	pinkish
